# Hidroxicloroquina para Pacientes com COVID-19 não Hospitalizados: Uma Revisão Sistemática e Metanálise de Ensaios Clínicos Randomizados

**DOI:** 10.36660/abc.20220380

**Published:** 2023-03-27

**Authors:** Rosa Lucchetta, Jessica Y. Matuoka, Haliton Alves de Oliveira, Gustavo Oliveira, Alexandre Biasi Cavalcanti, Luciano Azevedo, Otavio Berwanger, Renato Delascio Lopes, Regis Goulart Rosa, Viviane Cordeiro Veiga, Álvaro Avezum

**Affiliations:** 1 Hospital Alemão Oswaldo Cruz São Paulo SP Brasil Hospital Alemão Oswaldo Cruz, São Paulo, SP – Brasil; 2 Instituto Dante Pazzanese de Cardiologia São Paulo SP Brasil Instituto Dante Pazzanese de Cardiologia, São Paulo, SP – Brasil; 3 Hospital do Coração São Paulo SP Brasil Hospital do Coração, São Paulo SP – Brasil; 4 Hospital Sírio-Libanês São Paulo SP Brasil Hospital Sírio-Libanês, São Paulo, SP – Brasil; 5 Hospital Israelita Albert Einstein São Paulo SP Brasil Hospital Israelita Albert Einstein, São Paulo, SP – Brasil; 6 Duke University Hospital Durham North Carolina EUA Duke University Hospital, Durham, North Carolina – EUA; 7 Hospital Moinhos de Vento Porto Alegre RS Brasil Hospital Moinhos de Vento, Porto Alegre, RS – Brasil; 8 Beneficência Portuguesa de São Paulo São Paulo SP Brasil Beneficência Portuguesa de São Paulo, São Paulo, SP – Brasil

**Keywords:** COVID-19/tratamento farmacológico, SARS-CoV-2, Hidroxicloroquina, Ensaios Clínicos Controlados Aleatórios como Assunto, Metanálise

## Abstract

**Fundamento:**

Revisões sistemáticas anteriores não identificaram benefício do uso da hidroxicloroquina ou da cloroquina em pacientes com COVID-19 não hospitalizados. Após a publicação dessas revisões, os resultados do COPE, o maior ensaio clínico randomizado até hoje, tornaram-se disponíveis.

**Objetivos:**

Conduzir uma revisão sistemática e metanálise de ensaios clínicos randomizados (ECRs) para sintetizar as evidências sobre a eficácia e a segurança da hidroxicloroquina e da cloroquina em pacientes com COVID-19 não hospitalizados em comparação a controle ou tratamento padrão.

**Métodos:**

As buscas foram conduzidas nos bancos de dados PubMed, Embase, The Cochrane Library e ClinicalTrials.gov, e complementadas por busca manual. Foram realizadas metanálises diretas e avaliações de risco de viés e certeza da evidência, incluindo análise do tamanho ótimo da informação (OIS, *optimal information size*). Um nível de significância de 0,05 foi adotado na metanálise. PROSPERO: CRD42021265427.

**Resultados:**

Oito ECRs com 3219 participantes foram incluídos. As taxas de internação por COVID-19 e de eventos adversos não foram significativamente diferentes entre hidroxicloroquina (5,6% e 5,1%) e controle (7,4% e 20,4%) [risco relativo (RR) 0,77, intervalo de confiança 95% (IC95%), 0,57-1,04, I^2^: 0%; RR 1,78, IC95% 0,90; 3,52, I^2^: 93%, respectivamente)]. O OIS (7880) não foi alcançado para hospitalização por COVID-19, independentemente da simulação para a taxa de evento e redução do RR estimados.

**Conclusão:**

A evidência de muito baixa qualidade indicou falta de benefício com hidroxicloroquina em prevenir internações por COVID-19. Apesar de ser a revisão sistemática com o maior número de participantes incluídos, o OIS, considerando a resposta à infecção anterior à vacinação, não foi atingido.

**Figure:**
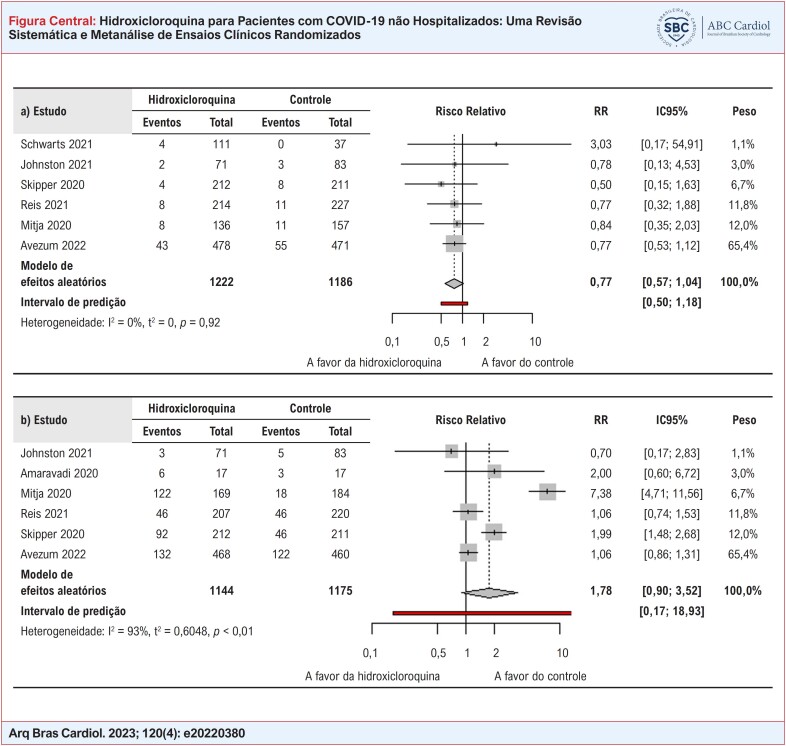
Forest plots a) internação por COVID-19 e b) quaisquer eventos adversos nos grupos hidroxicloroquina e controle. IC: intervalo de confiança, RR: Risco Relativo.

## Introdução

A pandemia da doença causada pelo novo coronavírus 2019 (COVID-19) ainda é um problema de saúde pública no mundo pelo elevado número de casos,^1^ devido à emergência de variantes tais como Alfa, Delta e Omicron,^2,3^ e o alto número de mortes,^1^ especialmente pelo reduzido acesso ou adesão à vacina e desenvolvimento de COVID-19 grave em indivíduos com fatores de risco cardiovasculares.^4–7^

Considerando o cenário da pandemia da COVID-19, várias opções terapêuticas foram adaptadas com base nos seus mecanismos de ação.^8^ A cloroquina e a hidroxicloroquina (HCQ), medicamentos antimaláricos, atuam nos mecanismos de afinidade do vírus SARS-CoV-2 com a enzima conversora de angiotensina 2.^9,10^ Por isso, esses medicamentos foram propostos como possíveis opções terapêuticas para pacientes com COVID-19, não só no ambiente hospitalar, como na profilaxia e para pacientes não internados. Embora diversos estudos, independentemente do cenário nosocomial, mostraram que a cloroquina e a HCQ não apresentam benefícios, tanto na mortalidade como na hospitalização, com piores perfis de segurança, o foco sobre populações não hospitalizadas é ainda pouco discutido.^11–16^

Duas revisões sistemáticas avaliaram o efeito da cloroquina/HCQ em pacientes com COVID-19 não internados.^17,18^ Ambas não mostraram benefícios clínicos da HCQ como tratamento de pacientes com COVID-19 não internados. No entanto, após a publicação dessas revisões sistemáticas, os resultados do estudo COPE - *COVID-19 Outpatient Prevention Evaluation*,^19^ o maior estudo randomizado conduzido até hoje, se tornaram disponíveis. Assim, conduzimos uma revisão sistemática e metanálise de ensaios clínicos randomizados (ECRs) para fazer uma síntese das evidências sobre a eficácia e a segurança da HCQ e da cloroquina em pacientes com COVID-19 não hospitalizados com dados atualizados do estudo COPE,^19^ e apresentar o tamanho ótimo da informação (OIS, *optimal information size*) da evidência disponível.

## Métodos

A revisão sistemática foi conduzida de acordo com as recomendações da Cochrane Collaboration e descrita segundo o *Preferred Reporting Items for Systematic Reviews and Meta-Analyses* (PRISMA 2020).^20,21^ O protocolo encontra-se registrado no registro prospectivo internacional de revisões sistemáticas PROSPERO (CRD42021265427).

### Critérios de elegibilidade

Consideramos estudos que preencheram os seguintes critérios de inclusão:

**População** – pacientes adultos com suspeita ou diagnóstico confirmado de COVID-19 não hospitalizados.

**Intervenção e controle** - HCQ ou cloroquina (pílulas ou qualquer outro medicamento na forma sólida) em qualquer dosagem, em comparação a placebo ou padrão de tratamento.

**Desfechos** – Os desfechos primários foram hospitalização por COVID-19 e qualquer evento adverso, e os desfechos secundários foram mortalidade, admissão na Unidade de Terapia Intensiva (UTI), tempo para a alta hospitalar, necessidade de intubação orotraqueal, tempo de ventilação mecânica, descontinuação por eventos adversos, e eventos adversos graves. Estudos que não apresentaram resultados de nenhum dos desfechos de interesse foram excluídos; e

**Tipo de estudo** – ECRs independentemente do número de comparadores, tempo de seguimento, número de participantes incluídos, ou status de publicação (publicado ou não publicado com resultado disponível no registro NCT).

### Fontes de informação e estratégias de busca

As buscas eletrônicas foram conduzidas no PubMed, Embase e Cochrane Library sem restrição de idioma (até setembro de 2021). O banco de dados do registro de ensaios clínicos (Clinicaltrials.gov) também foi incluído na pesquisa (até setembro de 2021), restringindo por registros contendo resultados. As listas de referências dos estudos incluídos e das revisões também foram pesquisadas. Resultados do estudo COPE foram compartilhados pelos autores em setembro de 2021. As estratégias completas de busca são apresentadas no texto suplementar 1. Filtros validados para ECRs foram aplicados.^22,23^ A validação da estratégia de pesquisa foi realizada por meio de uma busca nas listas de referências das revisões que avaliaram HCQ ou cloroquina para pacientes com COVID-19 (texto suplementar 2).

### Processo de seleção

Os registros obtidos foram importados no EndNote X8^®^ para remoção de duplicatas, e em seguida para a plataforma Rayyan para a seleção dos estudos.^24^ Dois pesquisadores (JYM e RCL) efetuaram, de maneira independente, o rastreio de títulos e resumos dos estudos para identificar registros irrelevantes. Em uma segunda etapa, textos completos dos artigos foram avaliados independentemente pelos mesmos dois pesquisadores de acordo com os critérios de elegibilidade. Discrepâncias foram resolvidas por consenso ou consulta a um terceiro revisor (HAOJ).

Os dados coletados foram: características do estudo (identificação, NCT, acrônimo, perfil da população geral, critério diagnóstico da COVID-19, variáveis comparadas, intervenção adicional em um dos grupos, país e número de centros, financiamento, período de estudo, e tempo de acompanhamento); características dos participantes de acordo com as variáveis comparadas (idade, número de participantes por sexo, hipertensão, asma ou diabetes); desfechos e resultados. Resultados relatados por subgrupos populacionais não foram extraídos e resultados múltiplos relatados por desfecho para diferentes tempos ou diferentes definições de desfecho foram extraídos.

### Avaliação de risco de viés

A avaliação de risco de viés dos estudos incluídos foi conduzida por dois revisores independentes (JYM e RCL). As discrepâncias foram resolvidas por consenso ou consulta a um terceiro revisor (HAOJ), utilizando a ferramenta revisada da Colaboração Cochrane (*Risk of Bias assessment tool for RCT*, RoB 2.0).^25^ Com base no risco de viés, o estudo poderia ser descrito como de “baixo risco” “com algumas preocupações" e “alto risco”. A avaliação foi conduzida tanto do estudo como dos desfechos primários.

A análise do risco de viés foi apresentada como um gráfico de semáforo dos julgamentos por domínio de cada desfecho, utilizando o *web app* RobVis.^26^

### Medidas dos efeitos, método de síntese e descrição dos vieses

O tamanho do efeito foi avaliado para cada desfecho por meio das medidas: razão de risco (RR) para desfechos dicotômicos (i.e., internação por COVID-19, eventos adversos, mortalidade, admissão na UTI, necessidade de intubação orotraqueal, descontinuação por eventos adversos e eventos adversos graves), e diferença média para os desfechos contínuos (i.e., tempo para a alta hospitalar e tempo de ventilação mecânica). Todas as medidas foram calculadas considerando um nível de significância de 0,05, intervalo de confiança de 95% (IC95%) e intervalo de predição.

Todos os estudos que preencheram os critérios de elegibilidade foram incluídos para a síntese narrativa. Para a síntese quantitativa, todos os estudos que apresentaram número de participantes com evento, número total de participantes com desfechos dicotômicos ou média de tempo e desvio-padrão (ou intervalo de confiança ou erro-padrão) para os desfechos contínuos foram considerados elegíveis. Se necessário, seria realizada conversão dos dados (por exemplo, intervalo de confiança para desvio padrão).

As análises estatísticas foram realizadas usando os pacotes meta e metafor do software R (R v4.1.2 and R studio 2021.09.0).^27–29^ Análises de heterogeneidade foram realizadas comparando-se populações, intervenções, controle e definições dos desfechos entre os estudos incluídos nas metanálises.

Metanálises diretas para os desfechos dicotômicos foram feitas usando o método Mantel-Haenszel, modelo aleatório, método de DerSimonian e Laird para o cálculo de tau^2^, método de Mantel-Haenszel para o cálculo de Q e tau^2^, e correção de continuidade de 0,5 em estudos com frequência zero de células foram empregados nas análises. Além da análise quantitativa da heterogeneidade clínica e metodológica dos estudos, análise estatística de inconsistência (I^2^) conforme proposto por Higgins e Green.^30^

A entrada dos dados foi realizada com base em contraste, isto é, dados a nível do ensaio em vez de dados a nível dos braços de intervenção. Para metanálises incluindo ensaios com múltiplos braços (mais de dois braços), três análises foram realizadas: i) seleção de um par de intervenções e exclusão dos demais (caso-base); combinação dos grupos para criar uma única comparação por pares (análise de sensibilidade), e metanálise de rede (análise de sensibilidade).^20^ As metanálises de rede foram conduzidas pela abordagem frequentista usando a plataforma MetaInsight.^31^

Outras análises de sensibilidade incluíram a remoção dos estudos com alto risco de viés, uso de uma metanálise alternativa (isto é, uso de modelo fixo em vez de modelo aleatório ou ajuste do modelo de efeitos aleatórios pelo método de Hartung-Knapp e Jonkman para cálculo do tau^2^), e método de retirada de um estudo por vez (*leave-one-out*).

Embora as análises de subgrupos, metarregressão e viés de publicação haviam sido planejadas no protocolo do estudo, elas não foram realizadas pelo fato de os critérios mínimos não haverem sido preenchidos (alta heterogeneidade estatística, relato de subgrupos comuns, relato de variáveis a nível do estudo, mínimo de 10 estudos, tamanhos amostrais diferentes e estimativas do efeito).

### Avaliação de certeza e tamanho ótimo de informação (OIS)

A avaliação de certeza foi realizada por dois revisores independentes (JYM e RCL) e discrepâncias foram resolvidas por consenso ou consulta a um terceiro revisor (HAOJ). A certeza da evidência foi avaliada usando o sistema GRADE (*Grading of Recommendations Assessment, Development and Evaluation*) para os desfechos primários (isto é, internação por COVID-19 e quaisquer eventos adversos), e classificada como “alta”, “moderada”, “baixa” e “muito baixa”.^32^ Assumiu-se o controle como o comparador comum, que poderia incluir placebo ou nenhum tratamento/padrão de tratamento. Avaliações de certeza estão resumidas nas tabelas “Resumo dos Achados”.

Para avaliar o risco de viés, viés de publicação e heterogeneidade, os métodos descritos acima foram considerados. Em relação à evidência indireta, foram consideradas diferenças potenciais entre a evidência incluída nesta revisão e a questão primária desta revisão. Para avaliar imprecisão das metanálises, o OIS foi calculado para a internação por COVID-19.^33–35^ O OIS pode ser definido como a quantidade mínima de informação necessária em uma metanálise para gerar conclusões confiáveis sobre a intervenção. Para se estimar o OIS de cada desfecho, é necessário calcular o tamanho amostral, incluindo as taxas de evento nos grupos controle e intervenção.^33–35^ Na ocasião em que esta revisão sistemática foi conduzida, não existia evidência sugerindo benefício da HCQ e, por isso, assumiu-se que o medicamento poderia reduzir o risco de internação (isto é, Redução no Risco Relativo, RRR) em 15%, 20% e 25% em comparação ao grupo controle. O risco no grupo controle foi obtido do tempo de hospitalização. Assim, o OIS foi apresentado em um gráfico, de acordo com os diferentes RRR, considerando um alfa de 0,05 e beta de 0,10 (poder de 90%). Ainda, foi realizada uma análise sequencial de ensaios de três RRRs.

## Resultados

### Seleção e características dos estudos

**Figura 1:**
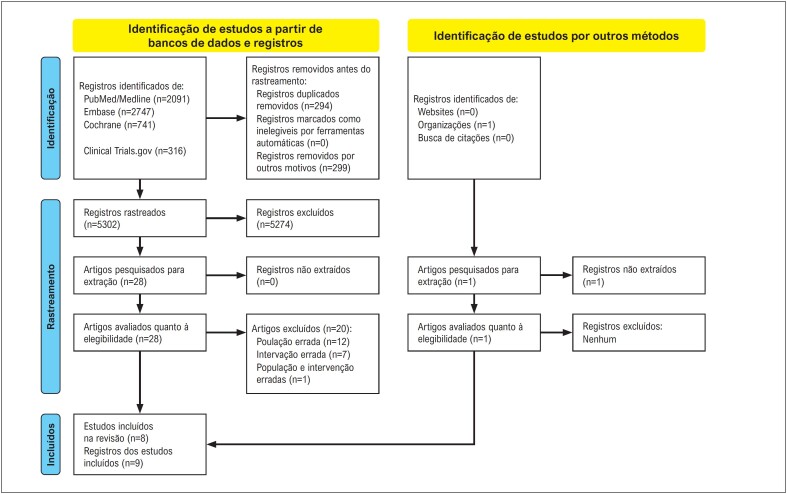
Fluxograma PRISMA. Page MJ, McKenzie JE, Bossuyt PM, Boutron I, Hoffmann TC, Mulrow CD, et al. The PRISMA 2020 statement: an updated guideline for reporting systematic reviews. BMJ 2021;372:n71. doi: 10.1136/bmj.n71. Para mais informações: http://www.prisma-statement.org/

Nossa busca identificou 5896 registros. Durante a avaliação de elegibilidade, 20 registros foram excluídos, por razões descritas no material suplementar 3. Após o processo de seleção (Figura 1), oito^12–16,19,36,37^ ECRs foram incluídos na revisão sistemática e metanálise.

Três estudos incluíram somente participantes com alto risco de complicações, no entanto, os fatores de risco variaram entre os estudos.^11–15,36^ A maioria dos estudos (n=6) incluiu apenas participantes com COVID-19 confirmado por reação em cadeia da polimerase da transcrição reversa em tempo real (rRT-PCR).^11–15,36^ No entanto, dois estudos que incluíram participantes com base no resultado do rRT-PCR e critérios adicionais (IgG, IgM, ou sintomas compatíveis) apresentaram 69,0%^16^ e 54,0%^19^ dos participantes com confirmação por rRT-PCR (Tabela 1).^16,19^ Uma vez que a maioria dos participantes incluídos nos estudos tiveram confirmação por rRT-PCR, escolhemos considerar somente uma população similar do ensaio COPE, isto é, uma população com intenção de tratar (ITT) modificada (ITTm) em vez da população com ITT em todas as metanálises. O estudo conduzido por Skipper et al.,^16^ que incluiu indivíduos com COVID-19, independentemente do método diagnóstico, foi incluído em todas as metanálises, mas excluído na análise *leave-one-out* para avaliar possível impacto da heterogeneidade da população. A maioria dos estudos recebeu financiamento (Tabela Suplementar 1).

**Tabela 1:** Características dos estudos incluídos na revisão sistemática em ordem decrescente de publicação

Estudo††	Perfil da população	Critérios diagnósticos para COVID-19	Country (N centers)	Study period (Follow-up time)
Avezum 2022 NCT04466540 COPE	Adultos não internados com suspeita ou diagnóstico confirmado de COVID-19 e ao menos um fator de risco para complicações clínicas	Confirmado (rRT-PCR ou IgM/IgG); suspeita (doença respiratória aguda e história de viagem ou transmissão comunitária ou contato com caso com um caso confirmado ou provável)	Brasil (56)	May 2020-July 2021 (30 dias)
Reis 2021 NCT04403100 TOGETHER	Pacientes ambulatoriais de alto risco†	rRT-PCR (swab nasofaríngeo)	Brasil (NR)	Junho-Setembro ou outubro 2020 (90 dias)
Johnston 2021 NCT04354428	Pacientes ambulatoriais adultos de baixo risco e de alto risco ‡	rRT-PCR (swab nasofaríngeo)	EUA (5)	Abril-Julho 2020 (28 dias)
Schwartz 2021 NCT04329611	Pacientes adultos com COVID-19 e pelo menos um fator de risco para doença grave	rRT-PCR (swab nasofaríngeo)	Canadá (NR)	Abril-Maio 2020 (30 dias)
Omrani 2020 NCT04349592 Q-PROTECT	Adultos com COVID-19 leve ou sem sintomas que se mantiveram em quarentena em Umm Qarn por não conseguirem realizar auto-quarentena	rRT-PCR (swab nasofaríngeo)	Qatar (2)	Abril-Agosto 2020 (21 dias)
Amaravadi 2020 (não publicado) NCT04329923 PATCH	Adultos (≥ 40 anos de idade) positivos para COVID-19 em isolamento em casa	rRT-PCR (swab nasofaríngeo)	EUA (1)	NR (até liberação da quarentena)
Mitja 2020 NCT04304053	Adultos com COVID-19 leve	rRT-PCR (swab nasofaríngeo)	Espanha (NR)	Março-Maio 2020 (28 dias)
Skipper 2020 NCT04308668	Adultos com COVID-19 leve	COVID-19 confirmado por teste laboratorial ou sintomas compatíveis e relação epidemiológica com um contato com COVID-19 confirmado por teste laboratorial	Canadá e EUA (NR)	Março-Maio 2020 (14 dias)

AZ: azitromicina, HCQ: hidroxicloroquina, NCT: National Clinical Trial (número); NR: não relatado; rRT-PCR: reação em cadeia da polimerase da transcrição reversa em tempo real; EUA: Estados Unidos da América

* - Idade > 65 anos; hipertensão; diabetes mellitus; asma; doença pulmonar obstrutiva crônica ou outras doenças pulmonares crônicas; tabagismo; imunossupressão; obesidade (definida como índice de massa corporal ≥ 30 Kg/m^2^; Idade 60 anos ou mais; doença pulmonar; hipertensão ou índice de massa corporal ≥ 30 Kg/m^2^;

† - Idade igual ou maior que 50 anos; presença de doença pulmonar, especificamente asma persistente grave ou moderada, doença pulmonar obstrutiva crônica, hipertensão pulmonar ou enfisema; diabetes que requer medicação oral ou insulina; hipertensão que requer tratamento; doenças cardiovasculares conhecidas [insuficiência cardíaca congestiva de qualquer etiologia, doença arterial coronariana documentada, doença cardíaca com manifestação clínica (miscelânea)]; doença pulmonar sintomática em tratamento crônico, história de transplantes, obesidade (índice de massa corporal ≥ 30 Kg/m^2^); estado de imunossupressão devido à doença; estado de imunossupressão devido a medicamento; pacientes com câncer;

‡ - Medicamentos determinados, terapias biológicas, condições médicas e outros fatores de risco ((i.e., idade ≥ 40 anos, índice de massa corporal > 40 Kg/m^2^, hipertensão (em tratamento clínico, tabagismo atual, transplante de medula óssea nos últimos 12 meses, transplante de órgãos sólidos, AIDS/HIV CD4 <200 nos últimos seis meses ou CD4>200 mas não em tratamento, linfopenia moderada (nos últimos seis meses: adultos <500), doença renal crônica, diabetes (em uso de hipoglicemiante ou insulina), doença arterial coronariana, insuficiência cardíaca/fração de ejeção ventricular esquerda, doença pulmonar crônica, asma, doença pulmonar intersticial, segundo diagnóstico médico), diagnóstico de câncer atual, deficiência imune congênita ou adquirida, cirrose;

†† todos os estudos adotaram um nível de significância de 0,05.

Todos os estudos avaliaram HCQ, a maioria comparando-a com placebo; dois estudos avaliaram HCQ e azitromicina e um estudo avaliou lopinavir + ritonavir (Tabela 2). A dose diária de HCQ (600-1200 mg) e duração do tratamento (5-14dias) variou entre os estudos (Tabela Suplementar 2). No total, 3219 participantes (mediana de 358; intervalo interquartil: 210-513 participantes por estudo) foram incluídos, dos quais 52,1% eram do sexo masculino (Tabela 2).

**Tabela 2:** Características dos participantes incluídos nos ensaios clínicos randomizados em ordem decrescente de publicação

Estudo	Alternativas comparadas*	n participantes (n homens)	Idade, mediana (DP) ou mediana (IIQ), anos	Qualquer doença coexistente, n (%)	Hipertensão, n (%)	Asma, n (%)	Diabetes, n (%)	Tempo desde o início dos sintomas até a inclusão, mediana (IIQ), dias
Avezum 2022	HCQ	478 (233)	47 (38-57)	466 (97,5)	278 (58,2)	51 (10,7)	89 (18,6)	4,0 (3,0-5,0)
	Placebo	471 (220)	48 (38-58)	456 (96,8)	261 (55,4)	57 (12,1)	74 (15,7)	4,0 (3,0-5,0)
Reis 2021	HCQ	214 (92)	53 (18-81)	NR	101 (47,2)	24 (11,2)	41 (19,1)	NR
	Lopinavir + ritonavir	244 (110)	54 (18-94)	NR	128 (52,5)	15 (6,1)	44 (18,0)	NR
	Placebo	227 (106)	53 (18-80)	NR	109 (48,0)	20 (8,8)	48 (21,1)	NR
Johnston 2021	HCQ + placebo	71 (32)	36 (19-78)	37 (52,1)	8 (11,3)	0 (0)	5 (7,0)	5,9 (4,0-7,8)
	HCQ+AZ	77 (30)	37 (18-71)	44 (57,1)	12 (15,6)	1 (1,3)	5 (6,5)	5,8 (3,9-8,3)
	Placebo	83 (38)	38 (18-70)	48 (57,8)	7 (8,4)	1 (1,2)	7 (8,4)	5,9 (4,0-8,3)
Schwartz 2021	HCQ	111 (65)	47 (12)	NR	29 (26,1)	12 (10,8)	18 (16,2)	7,0 (5,0-8,0)
	Placebo	37 (17)	47 (11)	NR	12 (32,4)	8 (21,6)	11 (29,7)	6,0 (6,0-9,0)
Omrani 2020	HCQ	152 (149)	40 (31-47)	NR	NR	NR	NR	NR
	HCQ+AZ	152 (150)	42 (38-48)	NR	NR	NR	NR	NR
	Placebo	152 (150)	41 (31-47)	NR	NR	NR	NR	NR
Amaravadi 2020	HCQ	17 (5)	56 (43-77)	NR	NR	NR	NR	NR
	Placebo	17 (8)	49 (40-80)	NR	NR	NR	NR	NR
Mitja 2020	HCQ	136 (32)	41 (12)	71 (52,2)	20 (14,7)	7 (5,1)	11 (9,0)	3,0 (2,0-4,0)
	Nenhum tratamento	157 (54)	42 (13)	85 (54,1)	15 (9,6)	10 (6,4)	9 (6,6)	3,0 (2,0-4,0)
Skipper 2020	HCQ	212 (89)	41 (33-49)	72 (34,0)	23 (10,8)	28 (13,2)	8 (3,8)	NR
	Placebo	211 (96)	39 (31-50)	64 (30,3)	23 (10,9)	20 (9,5)	7 (3,3)	NR

AZ: Azitromicina, HCQ: Hidroxicloroquina, IIQ: Intervalo Interquartil, NR: não relatado, n: número, DP: desvio padrão.

* Características do grupo intervenção e do grupo controle são descritos em detalhe no material suplementar (p 11)

### Risco de viés nos estudos

Os resultados da análise do risco de viés dos ECRs são apresentados na Figura Suplementar 1, Tabela Suplementar 3e Tabela Suplementar 4. Para a internação por COVID-19, houve predominância de “baixo risco” de viés (n=4 estudos), seguido de "com algumas preocupações" (n=3) e “alto risco de viés” (n=1) devido a limitações no processo de randomização, desvios das intervenções planejadas e seleção do resultado relatado.

Para o desfecho “qualquer evento adverso”, houve predominância de risco "com algumas preocupações" (n=3 estudos), seguido de “baixo risco” (n=20 e “alto risco” (n=1) devido a limitações em todos os domínios (isto é, processo de randomização, desvios das intervenções planejadas, dados de desfecho faltantes, medida do desfecho, e seleção do resultado relatado).

### Resultados dos estudos individuais e síntese

Mortalidade (n=8 estudos),^11–16,19,36^ eventos adversos graves (n=8),^11–16,19,36^ qualquer evento adverso (n=7),^11,12,14–16,19,36^ e internação por COVID-19 (n=6) ^11,12,14–16,19^ foram os desfechos comumente relatados entre os ECRs, seguidos por necessidade de intubação orotraqueal (n=3),^12,15,19^ admissão na UTI (n=2)^15,19^ descontinuação no tratamento por eventos adversos (n=2),^11,14^ e tempo de ventilação mecânica (n=2).^12,19^ O tempo para alta hospitalar não foi relatado por nenhum estudo. Embora oito ECRs tenham relatado mortalidade e eventos adversos graves, somente três estudos relataram um ou mais óbitos,^14,16,19^ e somente cinco relataram um ou mais pacientes com evento adverso sério;^12,14,15,19,36^ e, assim, contribuíram para a metanálise. Não foram conduzidas metanálises para o tempo de ventilação mecânica e necessidade de intubação orotraqueal, já que somente um estudo relatou um tempo superior a zero.^19^

Em relação à hospitalização por COVID-19, não se observou benefício estatisticamente significativo para o uso de HCQ nem em estudos individuais nem na metanálise (Figura Central). Identificou-se consistência estatística nesta metanálise, embora inconsistências clínica e metodológica tenham sido encontradas por comparação dos participantes (por exemplo confirmação por rRT-PCR, comorbidades dos participantes, dose, e duração do tratamento) e características dos estudos (por exemplo, tempo de acompanhamento e risco de viés). Ainda, não se observou benefício quanto à mortalidade, admissão da UTI, necessidade de intubação orotraqueal e tempo de ventilação mecânica (Tabela Suplementar 5 e Figura Suplementar 2).

Quanto aos eventos adversos, não houve prejuízos com a utilização de HCQ na metanálise (Figura Central), apesar de dois ECRs haverem descrito um maior risco de eventos adversos no grupo HCQ.^12,16^ Nesse caso, além da heterogeneidade clínica e metodológica, também foi identificada heterogeneidade estatística. A ausência de um efeito prejudicial com o uso da HCQ foi corroborada por desfechos secundários de segurança (Tabela Suplementar 5 e Figura Suplementar 2).

Todas as análises de sensibilidade dos desfechos primários foram consistentes com os achados das análises principais (Tabela Suplementar 6), exceto pela metanálise de “quaisquer eventos adversos” quando se assumiu um modelo fixo [RR 1,70 (IC95% 1,48 - 1,96), valor p < 0,0001) em vez de um modelo aleatório [RR 1,78 (IC95% 0,90 – 3,52), valor p = 0,10].

Vale ressaltar que, para o desfecho hospitalização por COVID-19, somente seis estudos foram incluídos na metanálise, já que outros dois incluídos na revisão sistemática não descreveram de forma clara se o desfecho avaliado foi internação por todas as causas ou somente por COVID-19. Contudo, mesmo com a inclusão desses dois estudos na análise de sensibilidade, a ausência do benefício da HCQ se manteve (Tabela Suplementar 6). Ainda, os resultados dos desfechos primários continuaram inalterados com a análise de sensibilidade do método *leave-one-out*, sugerindo que a heterogeneidade identificada qualitativamente não foi suficiente para afetar os resultados (Tabela Suplementar 6).

### Certeza da evidência

**Figura 2:**
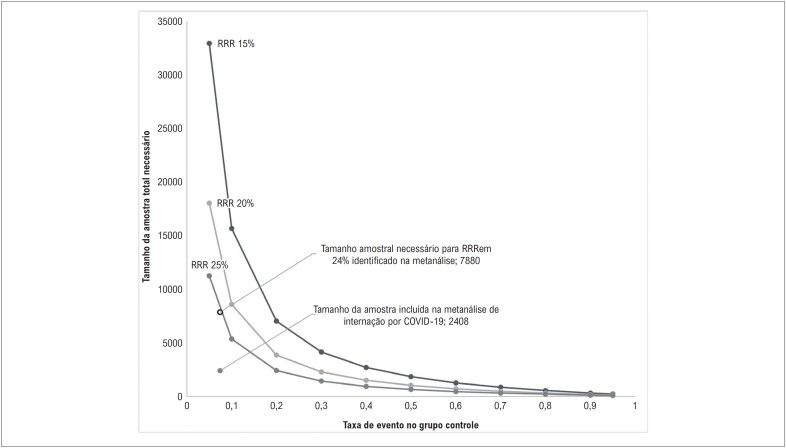
Tamanho ótimo de informação para diferentes estimativas de internação por COVID-19 entre os grupos. Alfa = 0,05, beta = 0,10 (Poder 90%); RRR: redução no risco relativo.

Para a hospitalização por COVID-19, a certeza da evidência foi classificada como “muito baixa”, uma vez que a heterogeneidade e a imprecisão dos domínios foram reduzidas em dois níveis, respectivamente (Tabela 3). A imprecisão foi responsável pela redução em dois níveis na certeza, já que para a diferença na hospitalização por COVID-19 identificada nesta metanálise (HCQ vs. controle: 5,6% vs 7,4%, p = 0,09), uma população aproximadamente três vezes maior (7,880) seria necessária par identificar qualquer diferença significativa (Figura 2). Assim, o OIS não foi alcançado. Uma ilustração da análise sequencial da hospitalização por COVID-19 está disponível na Figura Suplementar 4.

**Tabela 3:** Avaliação de certeza (GRADE) – Hidroxicloroquina comparada à terapia padrão para tratamento ambulatorial de COVID-19

Avaliação de certeza							Resumo dos achados				
Participantes (estudos) Acompanhamento	Risco de viés	Inconsistência	Evidência indireta	Imprecisão	Viés de publicação	Certeza da evidência	Taxas de eventos (%)		Efeito relativo (IC95% )	Efeitos absolutos previstos	
							Risco com terapia padrão	Diferença do risco com hidroxicloroquina		Risco com terapia padrão	Diferença do risco com hidroxicloroquina
Internação por Covid-19											
2408 (6 ECRs)	Não grave	grave*	Não grave	Muito grave†	Não há	⊕○ ○○ Muito baixa	88/1186 (7,4%)	69/1222 (5,6%)	**RR 0,77** (0,57 - 1,04)	74 por 1000	**Menos 17 a cada 1.000** (de 32 menos para 3 mais)
Quaisquer eventos adversos											
2,319 (6 ECRs)	Não grave	Muito grave*‡	Não grave	grave §	Não há	⊕○ ○○ Muito baixa	240/1175 (20,4%)	401/1144 (35,1%)	**RR 1,78** (0,90 – 3,52)	204 per 1,000	**Menos 159 a cada 1.000** (de menos 20 a mais 515)

IC: intervalo de confiança; RR: risco relativo.

* Heterogeneidade clínica e metodológica foi qualitativamente identificada (confirmação por rRT-PCR, comorbidades dos participantes, dose, duração do tratamento, tempo de seguimento e risco de viés).

† Para a diferença da internação por COVID-19 identificada nesta metanálise (hidroxicloroquina vs. controle: 5,6% vs. 7,4%), uma população aproximadamente três vezes maior que a incluída (tamanho ótimo de informação 7,880) deveria ser avaliada.

‡ Heterogeneidade estatística (I2: 93%).

§ O limite superior (RR 3,52) da metanálise é muito superior a RR 1,25, sugerindo imprecisão. O risco de viés não foi considerado um motivo para a redução do risco, devido ao maior peso dos estudos de “baixo risco de viés” na metanálise. No entanto, mesmo se o risco de viés fosse considerado grave, a certeza continuaria a mesma, “muito baixa”.

Para “qualquer evento adverso”, a certeza da evidência foi classificada como “muito baixa”, uma vez que a heterogeneidade e a imprecisão dos domínios (isto é, o limite superior do intervalo de confiança maior que RR1,25) foram reduzidos em dois e um nível, respectivamente (Tabela 3).

## Discussão

Esta revisão sistemática é a evidência científica mais abrangente e atualizada sobre o uso de HCQ no tratamento ambulatorial de pacientes com COVID-19, na prevenção de hospitalizações, incluindo oito ECRs e 3219 participantes. Mesmo incluindo o maior ECR, o ensaio COPE,^19^ esta metanálise sugere que não existe benefício significativo da HCQ, em comparação ao controle, na redução efetiva de internações por COVID-19 e outros desfechos de eficácia.

Resultados similares foram identificados em revisões sistemáticas anteriores com metanálises.^17,18^ Apesar de seu baixo valor em identificar e resumir a evidência disponível para essa população, esses estudos têm baixo poder estatístico para confirmar qualquer benefício potencial da HCQ na internação por COVID-19 e aplicaram diferentes abordagens metodológicas. Nossa revisão sistemática difere-se dessas anteriores por: i) avaliar o risco de viés no nível do estudo e no nível do desfecho conforme recomendado pelo RoB 2.0;^25^ ii) conduzir análises de sensibilidade para avaliar o impacto de estudos com alto risco de viés em vez de excluir os estudos não cegos; iii) conduzir análises de sensibilidade para avaliar o impacto de se excluir o terceiro braço de estudos com três braços em vez de se excluir todo o estudo; iv) conduzir a avaliação formal do OIS da internação por COVID-19, e v) incluir um maior número de ECRs e eventos.

Essas e outras escolhas metodológicas contribuíram para identificar que o risco de viés, a exclusão de um terceiro braço, e a heterogeneidade dos estudos não afetaram os resultados. Ainda, apesar da importância do estudo COPE em reduzir a imprecisão (RR 0,76 IC95% 0,45-1,28 para RR 0,77 IC95 0,57-1,04), o tamanho da amostra total ainda não é suficiente para confirmar ou refutar qualquer benefício da HCQ em diminuir internações por COVID-19, de acordo com o método do OIS.

Esse achado sugere que a baixa frequência de internações em adultos, independentemente do risco de complicações da COVID-19, e pequena diferença nas taxas entre HCQ e controles requerem amostras maiores para se confirmar qualquer benefício. Tal fato foi confirmado pela análise do OIS que sugere que, para se confirmar uma redução de 23% no risco de hospitalização por COVID-19 com o uso de HCQ, a evidência deveria incluir pela menos 7880 participantes, isto é, uma população três vezes maior que a disponível para esse desfecho. Ainda, os estudos incluídos na metanálise são anteriores à vacinação, quando se esperava um grupo controle com uma média de 7% de internação. Ao se considerar taxas de internação por COVID-19 muito menores para o grupo controle na fase atual da pandemia,^38^ o OIS seria bem maior que o estimado. Assim, a necessidade de amostras maiores, combinada com prioridades de pesquisa na fase pós-vacina, dificulta a realização de estudos maiores.

Além disso, nós identificamos uma alta heterogeneidade entre os estudos, considerando diferentes doses, frequência e duração do tratamento com HCQ, diferentes tempos de acompanhamento e inclusão somente de participantes com alto risco de complicações da COVID-19 por alguns estudos e de pacientes adultos independentemente do risco por outros. Consequentemente, houve proporções diferentes de participantes com comorbidades e idade média entre os estudos. Embora diferenças metodológicas e clínicas não tenham sido suficientes para modificar os resultados nas análises de sensibilidade, essa heterogeneidade não pode ser subestimada, uma vez que, para as metanálises incluindo estudos com intervalos de confiança amplos, a heterogeneidade estatística pode ser mascarada. Assim, nossa revisão sistemática identificou que a evidência disponível até o momento sugere a ausência potencial de benefício do uso de HCQ. Contudo, a certeza de tal evidência é muito baixa e, portanto, estudos maiores e bem conduzidos poderiam confirmar ou refutar com maior confiabilidade a eficácia e a segurança. Importante destacar que a avaliação da qualidade da evidência é subjetiva e, portanto, as explicações para a redução do risco de viés são apresentadas de modo que o leitor avalie sua confiabilidade.

Em relação à ausência de significância nos resultados sobre os efeitos negativos da HCQ, é importante considerar que a maioria dos estudos excluiu participantes com condições clínicas que possam aumentar o risco de eventos adversos sérios. Ainda, esta revisão sistemática não teve como objetivo identificar o risco do uso da HCQ para eventos adversos específicos, como arritmia. Vale ainda mencionar que, apesar de uma das análises de sensibilidade haver identificado um risco adicional para a incidência de “qualquer evento adverso” (modelo fixo), esse resultado não diminui a robustez da conclusão principal dessa revisão sobre ausência significativa de efeito negativo; o modelo fixo não é recomendado para metanálises com alta heterogeneidade, em que os intervalos de confiança tornam-se mais estreitos (maior precisão) e estudos influentes puxam a média ponderada para suas estimativas. Para esse desfecho, o estudo com alto risco de viés conduzido por Mitja et al. identificou um risco adicional para evento adverso com HCQ em comparação a nenhum tratamento (72% vs.10%).^12^

Embora haja fraca evidência sobre o uso de HCQ como profilaxia antes e após a exposição,^17,18^ tratamento ambulatoria^17,18^ ou hospitalar,^18,39^ este estudo corrobora os resultados de revisões publicadas sobre a falta de um benefício significativo da HCQ, independentemente da população avaliada. Assim, o uso de HCQ fora do contexto de pesquisa ainda não é recomendado.

Com base no movimento antivacina em todo o mundo,^4^ desigualdade no acesso à vacinação, o fato de que alguns indivíduos não desenvolvem imunidade efetiva após vacinação completa,^40^ e de que mesmos pessoas imunizadas possam necessitar de cuidado médico para COVID-19,^5^ investimentos em estudos que avaliam tratamentos para COVID-19 são necessários. Por outro lado, investimentos em estudos pequenos, com populações heterogêneas e sem validade metodológica devem ser desencorajados, uma vez que o maior potencial desses estudos é aumentar a incerteza, reduzir a credibilidade na ciência e contribuir para o uso irracional de tecnologias sem confirmação sobre seus riscos e benefícios. Assim, grandes estudos multicêntricos, com foco nos participantes com alto risco de complicações da COVID-19, com seguimento apropriado e suficiente, e elevada qualidade metodológica deveriam ser encorajados.

Algumas limitações da revisão sistemática devem ser mencionadas. Assim como em qualquer busca sistemática, a chance de estudos faltantes existe. Contudo, uma busca manual detalhada não identificou nenhum estudo na lista de referência de estudos relevantes. Devido aos desfechos avaliados e reportados no ECR, não conseguimos conduzir metanálises para todos os desfechos de eficácia identificados (isto é, tempo de ventilação mecânica, necessidade de intubação orotraqueal e tempo para alta hospitalar). No entanto, os achados atuais foram consistentes por todos os desfechos avaliados e, assim, há pouco potencial para diferentes resultados nesses desfechos não sintetizados na metanálise.

Há algumas correções à informação fornecida no protocolo (CRD42021265427): i) não foi especificado se o desfecho hospitalização referiu-se apenas às internações por COVID-19 ou às internações por todas as causas; e ii) não foi especificado que as análises de sensibilidade seriam feitas apenas para os desfechos primários (isto é, “hospitalização por COVID-19” e “quaisquer eventos adversos”. Os resultados sugerem que os achados seriam similares mesmo sem a implementação das modificações.

## Conclusões

Evidência de qualidade muito baixa mostrou falta de benefícios significativos do tratamento ambulatorial com HCQ na prevenção de hospitalização por COVID-19 em adultos com diagnóstico confirmado, o que foi corroborado por outros desfechos de eficácia avaliados (como mortalidade, admissão na UTI, tempo de ventilação mecânica, e necessidade de intubação orotraqueal). Considerando que os ECRs incluíram uma população selecionada, e que, por isso, podem não refletir as características da população geral que poderiam usar HCQ, não foram identificados prejuízos significativos na evidência atualmente disponível sobre “qualquer evento adverso” (certeza muito baixa), “evento adverso sério” ou “descontinuação por evento adverso”. Apesar de ser a revisão sistemática com o maior número de participantes incluídos, o OIS, considerando a resposta à infecção anterior à vacinação, não foi alcançado.

## * Material suplementar

Para informação adicional, por favor, clique aqui.
