# Supplementary material for: Hidroxicloroquina para Pacientes com COVID-19 não Hospitalizados: Uma Revisão Sistemática e Metanálise de Ensaios Clínicos Randomizados
Source: Arq Bras Cardiol. 2023 Mar 27;120(4):e20220380. [Article in Portuguese] doi: 10.36660/abc.20220380 (PMC10263429; doi:10.36660/abc.20220380)
Supplement: Translation

# Translation: Hydroxychloroquine for Non-Hospitalized COVID-19 Patients: A Systematic Review and Meta-Analysis of Randomized Clinical Trials

**Authors:** Rosa Lucchetta, Jessica Y. Matuoka, Haliton Alves de Oliveira, Gustavo Oliveira, Alexandre Biasi Cavalcanti, Luciano Azevedo, Otavio Berwanger, Renato Delascio Lopes, Regis Goulart Rosa, Viviane Cordeiro Veiga, Álvaro Avezum

**Affiliations:** 1 São Paulo SP Brazil Hospital Alemão Oswaldo Cruz, São Paulo, SP – Brazil; 2 São Paulo SP Brazil Instituto Dante Pazzanese de Cardiologia, São Paulo, SP – Brazil; 3 São Paulo SP Brazil Hospital do Coração, São Paulo SP – Brazil; 4 São Paulo SP Brasil Hospital Sírio-Libanês, São Paulo, SP – Brasil; 5 São Paulo SP Brazil Hospital Israelita Albert Einstein, São Paulo, SP – Brazil; 6 Durham North Carolina USA Duke University Hospital, Durham, North Carolina – USA; 7 Porto Alegre RS Brazil Hospital Moinhos de Vento, Porto Alegre, RS – Brazil; 8 São Paulo SP Brazil Beneficência Portuguesa de São Paulo, São Paulo, SP – Brazil

**Keywords:** COVID-19/drug therapy, SARS-CoV-2, Hydroxychloroquine, Randomized Controlled Trials as Topic, Meta-Analyis

## Abstract

**Background:**

Previous systematic reviews have identified no benefit of hydroxychloroquine and chloroquine in non-hospitalized COVID-19 patients. After publication of these reviews, the results of COPE, the largest randomized trial conducted to date, became available.

**Objectives:**

To conduct a systematic review and meta-analyses of randomized clinical trials (RCTs) to synthesize the evidence on the efficacy and safety of hydroxychloroquine and chloroquine for non-hospitalized COVID-19 patients compared to placebo or standard of care.

**Methods:**

Searches were conducted in PubMed, Embase, The Cochrane Library, and ClinicalTrials.gov complemented by manual search. Pairwise meta-analyses, risk of bias, and evidence certainty assessments were conducted, including optimal information size analysis (OIS). A level of significance of 0.05 was adopted in the meta-analysis. PROSPERO: CRD42021265427.

**Results:**

Eight RCTs with 3,219 participants were included. COVID-19 hospitalization and any adverse events rates were not significantly different between hydroxychloroquine (5.6% and 35.1%) and control (7.4% and 20.4%) (risk ratio, RR, 0.77, 95% confidence interval, CI, 0.57-1.04, I^2^: 0%; RR 1.78, 95%-CI 0.90; 3.52, I^2^: 93%, respectively). The OIS (7,880) was not reached for COVID-19 hospitalization, independently of the simulation for anticipated event rate and RR reduction estimate.

**Conclusion:**

Evidence of very low certainty showed lack of benefit with hydroxychloroquine in preventing COVID-19 hospitalizations. Despite being the systematic review with the largest number of participants included, the OIS, considering pre-vaccination response to infection, has not yet been reached.

**Figure:** Forest plots of a) COVID-19 hospitalization and b) any adverse events in hydroxychloroquine and control groups. CI: confidence interval, RR: risk ratio.

## Introduction

The coronavirus disease 2019 (COVID-19) pandemic is still a worldwide public health problem, given the high number of cases,^1^ due to the emergence of variants such as Alpha, Delta and Omicron,^2,3^ and the high number of deaths,^1^ especially due to reduced access or adherence to vaccine and development of severe COVID-19 among individuals with cardiovascular risk factors.^4–7^

Considering the COVID-19 pandemic scenario, several therapeutic options have been repurposed based on their respective mechanisms of action.^8^ The antimalarials chloroquine and hydroxychloroquine (HCQ) act on the affinity mechanisms of SARS-CoV-2 with the angiotensin-converting enzyme 2.^9,10^ For this reason, these drugs have been proposed as possible therapeutic options for patients with COVID-19, not only in the hospital setting, but also in prophylaxis and for non-hospitalized patients. Although several studies, regardless of the nosocomial scenario, have shown that chloroquine/HCQ does not present benefits, either in mortality or hospitalization, with worse safety profiles, the focus on the non-hospitalized population is still poorly discussed.^11–16^

Two systematic reviews evaluated the effect of chloroquine/HCQ in non-hospitalized COVID-19 patients.^17,18^ Both showed no clinical benefits of HCQ as treatment of non-hospitalized COVID-19 patients. However, after the publication of these systematic reviews, results of the COVID-19 Outpatient Prevention Evaluation (COPE),^19^ the largest randomized trial conducted to date, has become available. Thus, we aimed to conduct a systematic review and meta-analyses of randomized clinical trials (RCTs) to synthesize the evidence on the efficacy and safety of HCQ and chloroquine for non-hospitalized COVID-19 patients with updated data from the COPE Randomized Clinical Trial,^19^ providing the optimal information size (OIS) of the available evidence.

## Methods

The systematic review was performed in accordance with the Cochrane Collaboration recommendations and reported following the Preferred Reporting Items for Systematic Reviews and Meta-Analyses (PRISMA 2020).^20,21^ The protocol is registered in the International Prospective Register of Systematic Reviews (PROSPERO - CRD42021265427).

### Eligibility criteria

We considered studies that fulfilled the following inclusion criteria:

**Population** – Non-hospitalized adult patients with confirmed or suspected COVID-19.

**Intervention and control** – HCQ or chloroquine (pills or any other solid pharmaceutical form) in any dosage, compared to placebo or standard of care.

**Outcomes** – The primary outcomes were COVID-19 hospitalization and any adverse events; and the secondary outcomes were mortality, intensive care unit (ICU) admission, time to hospital discharge, need for orotracheal intubation, mechanical ventilation time, discontinuation due to adverse events, and severe adverse events. Studies that did not report results for any of the outcomes of interest were excluded; and

**Type of studies** – RCTs regardless of number of comparators, follow-up time, number of included participants, or report status (i.e., published, or unpublished studies if the result is available in the NCT registry).

### Information sources and search strategies

Electronic searches were conducted in the PubMed, Embase, and Cochrane Library without language restriction (until September 2021). Trial registration database (Clinicaltrials.gov) (until September 2021) was also searched, restricting to records containing results. Reference lists of reviews and included studies were also searched. Results of the COPE trial were shared by authors in September 2021. The complete search strategies are provided in the supplementary text 1. Validated filters for RCTs were applied.^22,23^ Validation of the search strategy was performed through a search in reference lists of reviews evaluating HCQ or chloroquine for patients with COVID-19 (supplementary text 2).

### Selection process

The retrieved registries were imported to EndNote X8^□^ for duplicate removal, then imported to Rayyan platform for study selection.^24^ Two researchers (JYM and RCL) independently screened the titles and abstracts of retrieved studies to identify irrelevant records. In a second step, full-text articles were also independently evaluated by the same two researchers according to the eligibility criteria. Discrepancies were resolved by consensus or by a third reviewer (HAOJ).

### Data collection process and data items

The data were independently extracted by two researchers (JYM, RCL) and discrepancies were reconciled in consensus meetings or using a third-party adjudication (HAOJ).

The collected data were study characteristics (identification, NCT, acronym, general population profile, COVID-19 diagnosis criteria, variables compared, cointerventions, country and number of centers, funding, study period, and follow-up time); participants’ characteristics according to compared alternatives (e.g. age, number of participants by sex, hypertension, asthma, or diabetes); outcomes and results. Results reported for population subgroups were not extracted, while multiple results reported per outcome for different time-points or different outcome definitions were extracted.

### Risk of bias assessment

Evaluation of risk of bias of the included studies was conducted by two independent reviewers (RCL, JYM). Discrepancies were resolved by consensus or consultation of a third reviewer (HAOJ), using the Cochrane Collaboration revised Risk of Bias assessment tool for RCT (RoB 2.0).^25^ Based on the risk of bias, the study could be described as ‘low risk’, ‘some concerns’ and ‘high risk’. The assessment was performed at the study and outcome level, considering the primary outcomes.

Analysis of the risk of bias analysis was presented as ‘traffic light plots’ of the domain-level judgements for each individual outcome, using RobVis web app.^26^

### Effect measures, synthesis methods, and reporting bias

Effect size measures were defined for each outcome as follows: risk ratio (RR) for dichotomous outcomes (i.e., COVID-19 hospitalization, any adverse events, mortality, ICU admission, need for orotracheal intubation, discontinuation due to adverse events, and severe adverse events) and mean difference for continuous outcomes (i.e., time to hospital discharge and mechanical ventilation time). All effect size measures were calculated considering a level of significance of 0.05, 95% confidence intervals (CI), and prediction interval.

All studies that met the eligibility criteria were eligible for narrative synthesis. For quantitative synthesis, all studies that reported the number of participants with event, number of total participants for dichotomous outcomes or mean time and standard deviation (or confidence interval or standard error) for continuous outcomes were eligible. If needed, data conversion (e.g., confidence interval to standard deviation) would be performed.

Statistical analyses were performed using meta and metafor R packages (R v4.1.2 and R studio 2021.09.0).^27–29^ Similarity analyses were performed by comparing population, interventions, control, and outcome definitions among the included studies in the meta-analyses.

Pairwise meta-analyses for dichotomous outcomes were made using the Mantel-Haenszel method, random model, DerSimonian-Laird estimator of tau^2^, Mantel-Haenszel estimator used in calculation of Q and tau^2^, and continuity correction of 0.5 in studies with zero cell frequencies were employed in all analyses. In addition to the qualitative analysis of methodological and clinical similarity of the studies, statistical analysis of inconsistency (I^2^) was performed as proposed by Higgins and Green.^30^

Data entry was performed with contrast-based data (i.e., trial-level summaries instead arm-level data). For meta-analyses including multi-arm trials (more than two arms), three analyses were performed: i) selection of one pair of interventions and exclusion of the others (base-case); combination of groups to create a single pair-wise comparison (sensitivity analysis), and network meta-analysis (sensitivity analysis).^20^ The network meta-analyses were conducted using MetaInsight platform by frequentist approach.^31^

Additional sensitivity analyses included the removal of studies at high risk of bias, use of an alternative meta-analysis (i.e., using fixed model instead random model or adjustment of the random effects model by the Hartung-Knapp and Jonkman method for calculation of tau^2^), and leave-one-out method.

Although analyses of subgroups, meta-regression, and publication bias have been planned in the review protocol, they were not performed due to non-compliance with the minimum criteria (high statistical heterogeneity, reporting of common subgroups, reporting of study-level variables, minimum of 10 studies, different sample sizes and effect estimates).

### Assessment of certainty assessment and optimal information size

Certainty assessment was conducted by two independent reviewers (RCJ, JYM) and discrepancies were reconciled in consensus meetings or using a third researcher as a referee (HAOJ). The certainty of the evidence was assessed using the Grading of Recommendations Assessment, Development and Evaluation (GRADE) System for the primary outcomes (i.e., COVID-19 hospitalization and any adverse events), and classified as ‘high’, ‘moderate’, ‘low’, and ‘very low’.^32^ Control was assumed as the common comparator, which could include placebo or no treatment/standard of care. Certainty assessments were summarized in the “Summary of Findings” Tables.

To assess the risk of bias, publication bias and heterogeneity, the methods described above were considered. Regarding indirect evidence, potential differences from the evidence included in relation to the guiding question of this review were considered. To assess imprecision of meta-analyses, OIS was calculated to COVID-19 hospitalization.^33–35^ The OIS can be defined as the minimum amount of information needed in a meta-analysis to draw reliable conclusions about an intervention. To estimate the OIS of each outcome, it is necessary to calculate the sample size including the event rates in the control and intervention groups.^33–35^ Since at the time this systematic review was conducted there was no evidence suggesting a benefit from HCQ, it was assumed that HCQ could reduce the risk of hospitalization (i.e., risk ratio reduction, RRR) by 15%, 20%, and 25% compared to the control group. The risk in the control group was obtained from the hospitalization meta-analysis. Therefore, OIS was presented as a plot, according to different RRR, considering an alpha of 0.05 and beta of 0.10 (Power 90%). Additionally, a trial sequential analysis of three RRR was carried out.

## Results

### Study selection and characteristics

Our search strategy identified 5,896 records. During eligibility step, 20 records were excluded, and the reasons are presented in supplementary text 3. After the selection process (Figure 1), eight RCTs^12–16,19,36,37^ were included in the systematic review and meta-analysis.

**Figure 1:** PRISMA flow chart. From: Page MJ, McKenzie JE, Bossuyt PM, Boutron I, Hoffmann TC, Mulrow CD, et al. The PRISMA 2020 statement: an updated guideline for reporting systematic reviews. BMJ 2021;372:n71. doi: 10.1136/bmj.n71. For more information, visit: http://www.prisma-statement.org/

Three studies included only participants with a high risk of complications, however, the risk factors varied among the studies.^11–15,36^ Most studies (n=6) included only participants with COVID-19 confirmed by real-time reverse transcription polymerase chain reaction (rRT-PCR).^11–15,36^ However, two studies that included participants based on rRT-PCR and additional criteria (e.g., IgG, IgM, or compatible symptoms) had 69.0%^16^ and 54.0%^19^ of participants with rRT-PCR confirmation (Table 1).^16,19^ Since most participants included in the studies had confirmation of COVID-19 by rRT-PCR, we chose to consider only a similar population of the COPE study, i.e., a modified intention-to-treat (ITT) mITT population of the COPE trial to the detriment of the ITT population in all meta-analyses. The study conducted by Skipper et al.,^16^ which includes individuals with COVID-19, regardless of diagnostic method, was included in all meta-analyses, but excluded in a leave-one-out analysis to assess the possible impact of population heterogeneity. Most studies received some funding (supplementary table 1).

**Table 1:** Characteristics of the studies included in the systematic review in descending order of publication

Study††	Population profile	COVID-19 diagnostic criteria	Country (N centers)	Study period (Follow-up time)
Avezum 2022 NCT04466540 COPE	Non-hospitalized adults with confirmed or suspected CO-VID-19 and at least one one risk factor for clinical complica-tions *	Confirmed (rRT-PCR or IgM/IgG); Suspected (Acute respiratory disease and travelling history or community transmission or contact with a confirmed or probable case)	Brazil (56)	May 2020- July 2021 (30 days)
Reis 2021 NCT04403100 TOGETHER	High-risk adult outpatients †	rRT-PCR (nasopharyngeal swab)	Brazil (NR)	Jun-Sep or Oct 2020 (90 days)
Johnston 2021 NCT04354428	Low-risk and high-risk outpatient adults‡	rRT-PCR (nasopharyngeal swab)	USA (5)	Apr-July 2020 (28 days)
Schwartz 2021 NCT04329611	Adults with COVID-19 with at least 1 risk factor for severe disease	rRT-PCR (nasopharyngeal swab)	Canada (NR)	Apr-May 2020 (30 days)
Omrani 2020 NCT04349592 Q-PROTECT	Adults with non-severe (mild or no symptoms) COVID-19 who were quarantined at Umm Qarn due to inability to self-quarantine	rRT-PCR (nasopharyngeal swab)	Qatar (2)	Apr-Aug 2020 (21 days)
Amaravadi 2020 (unpublished) NCT04329923 PATCH	Home bound COVID-19 positi-ve adults (≥40 years old)	rRT-PCR (nasopharyngeal swab)	USA (1)	NR (until quarantine release)
Mitja 2020 NCT04304053	Adults with mild COVID-19	rRT-PCR (nasopharyngeal swab)	Spain (NR)	Mar-May 2020 (28 days)
Skipper 2020 NCT04308668	Adults with early COVID-19	Laboratory-confirmed COVID-19 or COVID-19–compatible symptoms and an epidemiologic link to a contact with laboratory-confirmed COVID-19	Canada and USA (NR)	Mar-May 2020 (14 days)

AZ: azithromycin, HCQ: hydroxychloroquine, NCT: national clinical trial (number); NR: not reported; rRT-PCR: real-time reverse transcription polymerase chain reaction; USA: United States of America

* - Age > 65 years; hypertension; diabetes mellitus; asthma; chronic obstructive pulmonary disease or other chronic lung diseases; smoking; immunosuppression; obesity (defined as body mass index ≥ 30 Kg/m^2^; Age 60 years or greater; pulmonary disease; diabetes mellitus, hypertension, or self-reported body mass index ≥ 30 Kg/m^2^;

† - Age 50 years or older; presence of pulmonary disease, specifically moderate or severe persistent asthma, chronic obstructive pulmonary disease, pulmonary hypertension, or emphysema; diabetes requiring oral medication or insulin; hypertension requiring treatment; known cardiovascular diseases (congestive heart failure of any etiology, documented coronary artery disease, clinically manifested heart disease [miscellaneous]); symptomatic lung disease on chronic treatment; history of transplantation; obesity (body mass index ≥ 30 Kg/m^2^); immunocompromised status due to disease; immunocompromised status due to medication; and patients with cancer;

‡ - Determined medications, biologic therapies, medical conditions and other risk factor (i.e., Age 40 or over, body mass index > 40 Kg/m^2^, hypertension (on medical treatment), current cigarette smoker, bone marrow transplant within previous 12 months, solid organ transplant, AIDS/HIV CD4 <200 within last 6 months or CD4>200 but not on treatment, moderate lymphopenia (within previous 6 months: adults <500), chronic kidney disease, diabetes (on a hypoglycemic or insulin), coronary artery disease, heart failure/reduced left ventricular ejection fraction, chronic lung disease (chronic obstructive pulmonary disease, asthma, interstitial lung disease, as per physician diagnosis), any current cancer diagnosis, acquired or congenital immune deficiency, cirrhosis;

†† all studies adopted 0.05 of statistical significance.

All studies evaluated HCQ, most of them compared to placebo; two studies evaluated HCQ and azithromycin, and one study evaluated lopinavir + ritonavir (table 2). The daily dose of HCQ (600–1200 mg) and duration of treatment (5–14 days) varied between studies (Supplementary Table 2). In total, 3,219 participants (median of 358; interquartile range: 210-513 participants per study) were included, of which 52.1% were male (table 2).

**Table 2:** Characteristics of the participants included in the randomized clinical trials in descending order of publication

Study	Compared alternatives *	n participants (n men)	Age, median (SD) or median (IQR), years	Any coexisting disease, n (%)	Hypertension, n (%)	Asthma, n (%)	Diabetes, n (%)	Time from onset of symptoms to enrollment, median (IQR), days
Avezum 2022	HCQ	478 (233)	47 (38-57)	466 (97.5)	278 (58.2)	51 (10.7)	89 (18.6)	4.0 (3.0-5.0)
	Placebo	471 (220)	48 (38-58)	456 (96.8)	261 (55.4)	57 (12.1)	74 (15.7)	4.0 (3.0-5.0)
Reis 2021	HCQ	214 (92)	53 (18-81)	NR	101 (47.2)	24 (11.2)	41 (19.1)	NR
	Lopinavir + ritonavir	244 (110)	54 (18-94)	NR	128 (52.5)	15 (6.1)	44 (18.0)	NR
	Placebo	227 (106)	53 (18-80)	NR	109 (48.0)	20 (8.8)	48 (21.1)	NR
Johnston 2021	HCQ + placebo	71 (32)	36 (19-78)	37 (52.1)	8 (11.3)	0 (0)	5 (7.0)	5.9 (4.0-7.8)
	HCQ+AZ	77 (30)	37 (18-71)	44 (57.1)	12 (15.6)	1 (1.3)	5 (6.5)	5.8 (3.9-8.3)
	Placebo	83 (38)	38 (18-70)	48 (57.8)	7 (8.4)	1 (1.2)	7 (8.4)	5.9 (4.0-8.3)
Schwartz 2021	HCQ	111 (65)	47 (12)	NR	29 (26.1)	12 (10.8)	18 (16.2)	7.0 (5.0-8.0)
	Placebo	37 (17)	47 (11)	NR	12 (32.4)	8 (21.6)	11 (29.7)	6.0 (6.0-9.0)
Omrani 2020	HCQ	152 (149)	40 (31-47)	NR	NR	NR	NR	NR
	HCQ+AZ	152 (150)	42 (38-48)	NR	NR	NR	NR	NR
	Placebo	152 (150)	41 (31-47)	NR	NR	NR	NR	NR
Amaravadi 2020	HCQ	17 (5)	56 (43-77)	NR	NR	NR	NR	NR
	Placebo	17 (8)	49 (40-80)	NR	NR	NR	NR	NR
Mitja 2020	HCQ	136 (32)	41 (12)	71 (52.2)	20 (14.7)	7 (5.1)	11 (9.0)	3.0 (2.0-4.0)
	No treatment	157 (54)	42 (13)	85 (54.1)	15 (9.6)	10 (6.4)	9 (6.6)	3.0 (2.0-4.0)
Skipper 2020	HCQ	212 (89)	41 (33-49)	72 (34.0)	23 (10.8)	28 (13.2)	8 (3.8)	NR
	Placebo	211 (96)	39 (31-50)	64 (30.3)	23 (10.9)	20 (9.5)	7 (3.3)	NR

AZ: azithromycin; HCQ: hydroxychloroquine; IQR: interquartile range; NR: not reported; n: number; SD: standard deviation.

* The characteristics of intervention and control are reported in detail in supplemental material (p 11).

### Risk of bias in studies

Results of the risk of bias analysis of the RCTs are presented in the supplementary figure 1, supplementary table 3, and supplementary table 4. For COVID-19 hospitalization there was predominance of ‘low risk of bias’ (n=4 studies), followed by ‘some concerns’ (n=3), and ‘high risk of bias’ (n=1) due to limitations in ‘randomization process’, ‘deviations from the intended interventions’, and ‘selection of the reported result’.

For “any adverse event” outcome, there was predominance of ‘some concerns’ (n=3 studies), followed by ‘low risk of bias’ (n=2), and ‘high risk of bias’ (n=1) due to limitations in all domains (i.e., ‘randomization process’, ‘deviations from the intended interventions’, ‘missing outcome data’, ‘measurement of the outcome’, and ‘selection of the reported result’).

### Results of individual studies and synthesis

Mortality (n=8 studies),^11–16,19,36^ serious adverse events (n=8),^11–16,19,36^ any adverse events (n=7),^11,12,14–16,19,36^ and COVID-19 hospitalization (n=6)^11,12,14–16,19^ were the outcomes often reported among RCTs, followed by need for orotracheal intubation (n=3),^12,15,19^ ICU admission (n=2),^15,19^ discontinuation due to adverse events (n=2),^11,14^ and mechanical ventilation time (n=2).^12,19^ Time to hospital discharge was not reported in any study. Although eight RCTs reported mortality and serious adverse events, only three studies reported at least one death,^14,16,19^ and only five studies reported at least one patient with a serious adverse event;^12,14,15,19,36^ and, therefore, contributed to the meta-analyses. Meta-analyses were not conducted for mechanical ventilation time and need for orotracheal intubation, as only one study reported a time greater than zero.^19^

Regarding COVID-19 hospitalization, no statistically significant benefit for the use of HCQ was identified in individual studies, as well as in the meta-analysis (central figure). A statistical consistency was found in this meta-analysis, although clinical and methodological inconsistency has been identified by comparison of participants (e.g., rRT-PCR confirmation, comorbidities of participants, dose, and duration of treatment) and studies characteristics (e.g., follow-up time and risk of bias). Likewise, no benefit was observed for mortality, ICU admission, need for orotracheal intubation, and mechanical ventilation time (Supplementary Table 5 and Supplementary Figure 2).

Considering adverse events, no harm for the use of HCQ was identified in the meta-analysis (central figure), although two RCTs have reported a higher risk for adverse events in the HCQ group.^12,16^ In this case, in addition to the clinical and methodological heterogeneity, statistical heterogeneity was also identified. The absence of hydroxychloroquine harm was supported by secondary safety outcomes (Supplementary Table 5 and Supplementary Figure 2).

All sensitivity analyses of the primary outcomes were consistent with the findings of the main analyses (Supplementary Table 6), except for the meta-analysis of “any adverse events” when the fixed model was assumed (RR 1.70 [95%-CI 1.48; 1.96], p-value < 0.0001) instead of the random model (RR 1.78 [95%-CI 0.90; 3.52], p-value 0.10).

It is noteworthy that for COVID-19 hospitalization outcome only six studies were included in the meta-analysis, since another two included in the systematic review did not clearly describe whether the outcome evaluated was 'all-cause hospitalization' or 'COVID-19 hospitalization'. However, even with the inclusion of these two studies in the sensitivity analysis, the absence of HCQ benefit remained (Supplementary Table 6). In addition, results of primary outcomes remained unchanged with the sensitivity analysis of the leave-one-out method, suggesting that the qualitatively identified heterogeneity was not sufficient to affect the results (Supplementary Table 6).

### Certainty of evidence

For COVID-19 hospitalization the certainty of evidence was classified as ‘very low’, as the domains’ heterogeneity and imprecision were downgraded by one and two levels, respectively (table 3). Imprecision was responsible for the downgrade in certainty in two levels, as for the difference of COVID-19 hospitalization identified in this meta-analysis (HCQ vs. control: 5.6% vs 7.4%, p = 0.09) a population approximately 3 times greater (7,880) would be necessary to identify any significant difference (figure 2). Therefore, OIS was not reached. A figure showing trial sequential analysis for COVID-19 hospitalization is available in Supplementary Figure 4.

**Figure 2:** Optimal information size according to different estimates of COVID-19 hospitalization between groups. Alpha = 0.05, beta = 0.10 (Power 90%); RRR: risk ratio reduction.

**Tab1e 3:** Certainty assessment (GRADE) - Hydroxychloroquine compared to standard of care for outpatient treatment of COVID-1

Certainty assessment							Summary of findings				
Participants (studies) Follow-up	Risk of bias	Inconsistency	Indirectness	Imprecision	Publication bias	Overall certainty of evidence	Study event rates (%)		Relative effect (95%CI)	Anticipated absolute effects	
							With standard of care	With hydroxychloroquine		Risk with standard of care	Risk difference with hydroxychloroquine
**Covid-19** **hospitalization**												
2,408 (6 RCTs)	not serious	Serious *	not serious	very serious†	none	⊕○ ○○ Very low	88/1186 (7.4%)	69/1222 (5.6%)	**RR 0.77** (0.57 - 1.04)	74 per 1,000	**17 fewer per 1.000** (from 32 fewer to 3 more)
**Any adverse** **events**											
2,319 (6 ECRs)	not serious	very serious*‡	not serious	serious §	none	⊕○ ○○ Very low	240/1175 (20.4%)	401/1144 (35.1%)	**RR 1.78** (0.90 – 3.52)	204 per 1,000	**159 more per 1.000** (from 20 fewer to 515 more)

CI: confidence interval; RR: risk ratio; RCT: randomized clinical trial. Explanations:

* Clinical and methodological heterogeneity was qualitatively identified (rRT-PCR confirmation, comorbidities of participants, dose, duration of treatment, follow-up time, and risk of bias).

† For the difference of Covid-19 hospitalization identified in this meta-analysis (HCQ vs control: 5.6% vs 7.4%), a population of about 3 times greater than the one included (optimal information size 7,880) should be evaluated.

‡ Statistical heterogeneity (I2: 93%).

§ The upper limit (RR 3.52) of meta-analysis is much higher than RR 1.25, suggesting imprecision. The risk of bias was not considered a reason for downgrading because of the greater weight of 'low risk of bias' studies in the meta-analysis. However, even if the risk of bias was considered serious, the certainty would be "very low" just the same.

For ‘any adverse events’ the certainty of evidence was classified as ‘very low’, as the domains’ heterogeneity and imprecision (i.e., upper limit of the confidence interval greater than RR 1.25) were downgraded by two and one levels, respectively (Table 3).

## Discussion

This systematic review is the most updated comprehensive scientific evidence on HCQ as outpatient treatment for COVID-19 patients, for preventing hospitalization, including eight RCTs and 3,219 participants. Even including the largest RCT, the COPE trial,^19^ the meta-analysis suggests that there is no significant benefit in using HCQ, as compared to control, to effectively reduce COVID-19 hospitalizations and other related efficacy outcomes.

Similar results were identified by previous systematic reviews with meta-analyses.^17,18^ Despite their value in identifying and summarizing the evidence available for this population, these studies have low statistical power to confirm any potential benefit of HCQ in COVID-19 hospitalization and have applied different methodological approaches. Our systematic review differs from the previous ones by: i) assessing the risk of bias at the study and outcome level, as recommended by RoB 2.0;^25^ ii) conducting sensitivity analyses for assessing the impact of high risk of bias studies instead of excluding unblinded studies; iii) conducting sensitivity analyses to assess the impact of excluding the third arm of studies with three-arm rather than excluding the study; iv) conducting formal evaluation of OIS to COVID-19 hospitalization; and v) including greater number of RCTs and events.

These and other methodological choices contributed to identifying that the risk of bias and the exclusion of a third arm, or the heterogeneity of the studies did not affect the findings. In addition, despite the COPE trial importance for reducing imprecision (RR 0.76 95%-CI 0.45-1.28 to RR 0.77 95%-CI 0.57-1.04), the overall sample size is still not sufficient to confirm or refute any benefit of hydroxychloroquine in reduce COVID-19 hospitalizations, according to the OIS method.

This finding suggests that the low frequency of hospitalization in adults regardless of the risk for complications of COVID-19, and small difference in the rates between HCQ and controls, require larger sample sizes to confirm any potential benefit. This was confirmed by the OIS analysis which suggests that, to confirm a 23% reduction in the risk of COVID-19 hospitalization with the use of HCQ, the evidence should include at least 7,880 participants, i.e., a population three times greater than that available for this outcome. Additionally, the studies included in the meta-analysis were conducted in the pre-vaccination era, when the control group was expected to have, on average, 7% of hospitalization. When considering the much lower rates of hospitalization in the current phase of the pandemic for a control group,^38^ the OIS would be much higher than the estimated. Thus, the need for larger sample sizes, combined with post-vaccine research priorities makes it difficult to carry out larger studies.

We also identified a high heterogeneity between studies, considering different doses, frequency and duration of treatment with HCQ; different follow-up times; and the inclusion of only participants at high risk for COVID-19 complications by some studies and inclusion of adult participants regardless of risk by others. Consequently, there were different proportions of participants with comorbidities and mean age between studies. Although clinical and methodological differences were not sufficient to modify the findings in the sensitivity analyses, this heterogeneity should not be underestimated, since for meta-analyses including studies with wide confidence intervals the statistical heterogeneity can be masked. Therefore, our systematic review identified that the available evidence to date suggests the potential absence of benefit of using HCQ. However, it is of ‘very low certainty’ and, therefore, larger, and well conducted studies would confirm or refute reliably both efficacy and safety. It is important to highlight that the evaluation of the evidence quality is subjective and hence explanations for downgrade are presented so that the reader can judge how reliable the findings are.

Regarding the finding of no significant harm of using HCQ, it is important to consider that most studies excluded participants with clinical conditions that may increase the risk of serious adverse events. Furthermore, it was not the aim of this systematic review to identify the risk of HCQ for the incidence of specific adverse events (e.g., arrhythmia). It is also highlighted that, although in one of the sensitivity analyses an additional risk for the incidence of ‘any adverse events’ was identified (fixed model). This result does not diminish the robustness of the main conclusion of this review of no significant harm; the fixed-model is not recommended for meta-analyses with high heterogeneity, in which confidence intervals get narrower (greater precision) and influential studies pull the weighted mean towards their estimate. For that outcome, the high risk of bias study conducted by Mitja et al. identified an additional risk of adverse event with HCQ compared to no treatment (72% vs 10%).^12^

Although weak evidence is available on the use of HCQ as pre- and post-exposure prophylaxis,^17,18^ outpatient^17,18^ or inpatient treatment,^18,39^ this review supports the findings of published reviews on the lack of significant benefit of HCQ, independently of the assessed population. Therefore, the use of HCQ outside the research context persists not recommended.

In light of the anti-vaccine movement worldwide,^4^ global vaccine inequity, and the facts that certain individuals do not develop efficient immunity after full vaccination,^40^ and that even immunized people could need medical care for COVID-19,^5^ investments in studies that evaluate treatments for COVID-19 are absolutely needed. On the other hand, investing in small studies, with heterogeneous populations and without methodological validity should be discouraged, since the greatest potential of these studies is to increase uncertainty, reduce credibility in science and contribute to the irrational use of technologies without confirmation on their risks and benefits. Thus, large multicenter studies focused on participants at high risk for COVID-19 complications, with proper and sufficient follow-up, and high methodological quality should be encouraged.

Some limitations of our systematic review should be mentioned. As in any systematic search, the chance of missing studies exists. However, a careful manual search found no additional studies in the reference list of relevant studies. Due to the outcomes assessed and reported in RCT, we could not conduct meta-analyses for all efficacy outcomes identified (i.e., mechanical ventilation time, need for orotracheal intubation, and time to hospital discharge). Nevertheless, the current findings were consistent across all outcomes evaluated and, therefore, there is little potential for different results in these outcomes not synthesized by meta-analysis.

There are some amendments to information provided in the protocol (CRD42021265427): i) it was not specified whether the outcome hospitalization referred only to those caused by COVID-19, or to all-cause hospitalization; and ii) it was not specified that the sensitivity analyses would be performed only for the primary outcomes (i.e., ‘COVID-19 hospitalization’ and ‘any adverse events’). Findings suggest that similar results would be achieved even without implementing the modifications.

## Conclusions

Very low certainty evidence showed lack of significant benefits of outpatient treatment with HCQ in preventing COVID-19 hospitalization in adults with a confirmed diagnosis, which was corroborated by other efficacy outcomes evaluated (i.e., mortality, ICU admission, mechanical ventilation time, and need for orotracheal intubation). Considering that the RCTs included a selected population and that, therefore, may not reflect the characteristics of the general population that could use HCQ, no significant harm was identified in available evidence on “any adverse event” (very low certainty), serious adverse event, or discontinuation due to an adverse event. Despite being the systematic review with the largest number of participants included, the OIS, considering pre-vaccination response to infection, has not yet been reached.

## * Supplemental Materials

For additional information, please click here.
